# 

**Published:** 1921-08-13

**Authors:** 


### Army Dental Corps.

The War Office announces that the rates of half-pay for officers of the Army Dental Corps, which were fixed in January last at half the rates of full pay, have been amended, and will now be the rates provided for other arms of the Service in terms of Army Order 324 of 1919.

MESSRS. MELDRUMS, LTD., Timperley, Manchester, have been awarded a Bronze Medal at the Folkestone Meeting of the Sanitary Institute for their new portable "Sack" disinfector.

### Passing Events and Topics.

### Yeovil's New Hospital.

Colonel G. C. W. Heneage, D.S.O., has laid the foundation-stone of the new Yeovil and District Hospital, which will replace the institution founded fifty years ago. In addition to the accommodation in the old hospital, which received 242 in-patients last year, the new work now proceeding will provide two wards of fourteen beds apiece, four single rooms, and staff accommodation. Towards the cost of £17,900 a sum of over £14,000 is in hand. The complete scheme will cost £30,000, which cannot be taken in hand until funds permit.

### Boxing and Charity.

The world-wide interest shown in the recent fight between Carpentier and Dempsey in America is an indication that boxing holds its own with other sports in the estimation of the general public. If anything the interest seems to increase, or becomes more noticeable with the growth of population and of newspapers. How far, however, boxing tournaments may be of value to hospitals is uncertain. They are occasionally held for charitable objects. One was held last month, we understand, in aid of St. Thomas's Hospital, but, judging by a casual reference, the proceeds do not seem to have been large.

### University College Hospital.

When the educationists from the Dominions arrived in London, before attending the Congress of Universities of the Empire at Oxford, they visited University College Hospital. During their inspection it was explained that, through the generosity of the Rockefeller Trustees, it is hoped to give to the hospital an obstetric annexe and additional laboratories. When the buildings have been finished the medical school will possess three whole-time teachers of medicine, surgery, and obstetric medicine.

### A Hospital Cinema Film.

A cinema film illustrating what is done at the Prince of Wales's Hospital, Cardiff, especially in regard to the manufacture of artificial limbs, has been prepared, at the request of Sir David Henderson, for exhibition, under the auspices of the International Red Cross, in all the countries who are constituents of that organisation. This, it is expected, will lead to the foundation of hospitals in those lands, modelled on that at Cardiff.

### A Hospital's Loss Through the Strike.

A very interesting survey was made by Mr. William Johnson, J.P., Chairman of Royal Albert Edward Infirmary, Wigan, at the recent annual meeting. Of the income, which rose to £28,500 last year, half was contributed in weekly contributions, and nearly £3,000 more was raised from this source than in the preceding year. The entertainment tax robbed them of about a quarter of the £550 raised by the Fête, and Mr. Johnson protested against the application of the tax to such enterprises, where not only everyone's services are voluntary, but where many give money as well. The first year of the Ladies' Linen League has abundantly justified itself. Unfortunately the expenditure has increased more than the income, and overdue alterations to the kitchen and domestic offices have again to be postponed. But if the principle of 2d. a week could be accepted in all the works and factories of the town and district it would go far to solve the Infirmary's difficulties. Annual subscriptions have decreased, and Mr. Stanley Brunt, the Superintendent and Secretary, is to make a special effort to revive them. The Mayor, who presided, said that during the industrial crisis the infirmary lost fourteen weeks' contributions from the mine workers and from others indirectly affected, amounting in all to £8,000, so that they have to face a serious loss in this respect.

### Leicester Royal Infirmary.

Colonel Bond, Deputy-Chairman, presiding over the half-yearly meeting of the Governors, pointed out the immense advantage to the community to have an institution possessed of the *personnel* and the equipment to deal effectively and expeditiously with upwards of 6,000 accidents, severe and slight, in the period under review—accidents from the factory, the workshop, the street, the home. Lieut.-Colonel C. F. Oliver, V.D., has been appointed Treasurer to the institution in the place of Mr. Duncan Henderson. Mr. J. A. Hartopp announced that the Infirmary Sports—a fixture that had given great pleasure and brought considerable funds to the institution years ago—has been revived. In submitting their report the Governors referred to the continued high cost of maintenance. While some prices had been reduced others had considerably advanced, and there was undoubtedly a prospect of harder prices for some food supplies owing to the drought. The total expenditure for the period was £33,582, an advance of about £1,400 on the corresponding half-year. Receipts have realised £18,742; annual subscriptions are well maintained; and other receipts include £1,116 to the Children's Hospital and £733 to legacies. Since the accounts have been made up the institution has received a £1,000 legacy under the will of the late Mr. T. Fielding Johnson, J.P. (ex-Chairman), and £500 to the Children's Hospital, as well as £2,000 on account of the residuary estate of the late Mr. Edwin Carr. To commemorate the 150th anniversary year the "unit" system of contributing has been evolved. A "unit" is 12s. 6d., and represents a penny a year for each year's service. It is hoped that the public will subscribe one or more "units" according to the extent of their interest in city and county as judged by the rateability of their premises. In this way it is hoped to raise £100,000, and to secure for the institution the interest of residents who have never before contributed directly to the infirmary.

### The Welsh National School of Medicine.

At the first meeting of the Medical Board of the Welsh National School of Medicine, Cardiff, it was decided, after some discussion, to recommend the University Council to open the Final School in October next. Sir William Diamond, speaking as Chairman of King Edward VII. Hospital, said that, if the arrangements already made were not carried out, the University would place the hospital in a most invidious position. It had put everything in order at the urgent request of Cardiff College Council, and the hospital could not make similar provision for the students in a year's time. The difficulty is apparently one of finance, but, while prudence might suggest delay until all the necessary money has been raised, the original supporters of the scheme have to be considered, and a joint committee of the University Council, the University College Council, and King Edward VII. Hospital is to be formed to issue an appeal. There are over 200 medical students in Cardiff preparing for the opening of the full course. In October the medical school in Cardiff will provide the final courses of medical training and the means to qualify for medical degrees.

OWING to the strike of the commercial printers which has extended all over Canada since May 1, our contemporary, *The Canadian Nurse*, has not been issued since April. It is hoped to bring out the July number, and possibly subscription dates may be advanced two months so that no hardship is inflicted on subscribers.

### Founders' Day at Gloucester.

On July 23 a Fête was held in the grounds of the hospital to celebrate Founders' Day. The wards and various departments were open to visitors, and the grounds were gay with stalls and side-shows. The Cotswold Players gave two performances of a new historical play, "The Breaking Point," and the Wagon Works Silver Band delighted everyone with their fine selections. A wireless-telegraphy receiving apparatus provided a novel and interesting form of attraction. Teas were served in the nurses' garden. Although the briskness of the proceedings was somewhat marred by the uncertainty of the weather, the amount realised was no less than £210. Pound gifts to the value of £39 were taken at the gate. The primary object of the organisers—viz., to create and preserve a widespread personal interest in the hospital—is undoubtedly greatly furthered by such a practical celebration of Founders' Day.

### The League of Remembrance.

The first anniversary of the League of Remembrance, which does such useful work in making articles for hospitals and Infant Welfare Centres, was celebrated by a dinner at the Connaught Rooms. Princess Beatrice, the Duchess of Albany, and Princess Marie Louise honoured the gathering with their presence. The Chairman, Mr. John Murray, C.V.O., in his speech told how the League grew from the War Depôt in Cavendish Square, largely on the initiative of H.R.H. the Duchess of Albany, who desired to make permanent the devotion and energy that had found vent during the war. The present work of the League is described in The Hospital of July 2. During the last year 200,000 articles have been made for hospitals and other institutions in its home at 2 Marlborough Gate. As the League makes up free of charge all materials sent to it to any desired pattern, it is a real saving to hospitals, both in money and in time. The League at present numbers about 1,200 members, but its usefulness could be largely extended by an increased membership.

### Answers to Correspondents.

*(Correspondents are reminded that all inquiries are answered in these columns. We do not give postal replies. A coupon cut from the current issue must be enclosed with each inquiry.)*

Posts in a School or Institution.—Your best plan is to watch the advertisements in The Hospital, *The Nursing Mirror*, and the other nursing papers; also in the daily papers. We note you and your friend want to find a post together as matron and assistant matron or nurse, both being fully trained, and that you want to have your private sitting-room. You may find it quicker to advertise than to wait for such vacancies to be advertised. A cottage hospital might answer your requirements. It would only be the larger schools where two trained nurses would be employed.—F. M. C.

Massage Training.—The minimum time for training for massage is twelve months. Write for particulars and fees to the Secretary, Chartered Society of Massage and Medical Gymnastics, 157 Great Portland Street, W. You would have to give up the greater part of your time to the work whilst training, and would not be able easily to continue other occupation.—B. S.

Nursing Work in Hospitals in China or India.—As you are untrained we do not see what employment you could get. If you want to train as a nurse there is the Government General Hospital, Madras, or the Presidency General Hospital, Calcutta. Perhaps one of these might receive you, but you would probably have to train alongside of Eurasians. In China there is the Shanghai General Hospital or the St. Luke's Hospital. Write to the matrons in each case.—G. B.

Nursing Books in General and Maternity Work.—"The Handbook for Nurses," by J. K. Watson, 11s. 3d. net, post free; "The Practical Text-Book of Midwifery," by Robert Jardine, 8s. 3d. net, post free; "A Complete Handbook of Midwifery," by J. K. Watson, 11s. 3d. net, post free. You can obtain all these from The Scientific Press, 28 and 29 Southampton Street, Strand, W.C.—M. G. H.

Books on Elementary First-Aid and Home Nursing.—"The British Red Cross Society First-Aid and Home Nursing, Manuals I. and II.," by Sir James Cantlie; "Training in First-Aid and Nursing by Question and Answer," by Capt. S. T. Beggs, M.D., 2s. 2d., post free; "Home Nursing for the Use of Red Cross Workers and in the Home," by Edith Newsome, 2s. 9d., post free. You can obtain all these from The Scientific Press, 28 and 29 Southampton Street, Strand, W.C.—E. M. W.

### Garden Fete at Cardiff.

On Saturday, July 23, a very successful garden fête was held in the grounds of Greenlawn, Cardiff (by kind permission of Sir John Lynn-Thomas, K.B.E., C.B., C.M.G.), in aid of the Prince of Wales' Hospital for Limbless and Cripples, Cardiff. Two Cardiff gentlemen undertook to defray the expenses, so that the whole of the gross proceeds might go to the hospital. In addition to the usual features associated with a garden fête, there were several novelties that added interest to the function and assisted the funds considerably. Two signed photographs of the Prime Minister and Dame Margaret Lloyd George were put up to auction and secured 50 guineas, the purchaser being Sir William Diamond, who has always been a generous supporter of the hospital. A live young pig was sold, with the condition that the purchaser should lead it home. The result was that the pig was resold several times, realising £10, the final purchaser handing it over to Major Linthune, of Rookwood Hospital, for the benefit of the patients there. Other profitable sales were an average price of 5s. each for twenty bananas. With such keen buying, it is not surprising that, despite threatening skies, the amount realised by the fête was £500.

### Matrons' and Sisters' Appointments.

Royal Eye and Ear Hospital, Bradford.—Miss Ella Mallinson has been appointed theatre sister and assistant matron. She was trained at the General Hospital, Altrincham, and has been successively staff nurse and sister to the children's ward at the Royal Eye and Ear Hospital, Bradford.

County Sanatorium, Branston Hall, Lincoln.—Miss J. T. Ruddy has been appointed matron. She was trained at the Royal Salop Infirmary, and has been matron of the Isolation Hospital and Women's Sanatorium, Stone.

West Bromwich and District Hospital.—Miss A. Blakey has been appointed night sister and Miss E. Teal theatre and casualty sister. The former was trained at Preston Royal Infirmary, where she was later sister of the children's ward, temporary night sister, and on the private nursing staff. Miss Teal was trained at the Royal West Sussex Hospital and at the City of London Maternity Hospital; she served in the T.F.N.S. at home and abroad for four years, and has done private nursing.

Hospital for Women, Liverpool.—Miss E. F. Potter has been appointed theatre sister. She was trained at the Coventry and Warwickshire Hospital, has been ward sister at the Chester War Hospital, and night sister at the Hospital for Women, Liverpool.

Wingrove Hospital, Newcastle-on-Tyne.—Miss Mary C. Taylor has been appointed assistant matron and Miss I. Russell home sister. The former was trained at the Glasgow Western Infirmary, has been matron of the Children's Home, Scotstoun, and assistant matron of the Leith Hospital; she was on military service (Q.A.I.M.N.S.R.) for four and a-half years. Miss Russell was trained at Lambeth Infirmary; she has been sister-in-charge of the Infirmary Cottage Homes, Marston Green, near Birmingham, and night sister at the Royal Infirmary, Aberdeen. She served in the Territorial Force Nursing Service as night sister.

Gateshead Union Infirmary.—Miss Elsie Fletcher has been appointed superintendent nurse. She was trained at Mill Road Infirmary, Liverpool, and has been superintendent nurse of Bedwelty Union.

Glasgow Royal Infirmary.—Miss M. Stewart Donaldson has been appointed matron. She was trained at the Great Northern Central Hospital, and has been matron of Mount Vernon Hospital for Consumption, Units in Serbia and France, and of the London Temperance Hospital.

Portsmouth Workhouse Infirmary.—Miss C. M. Priest has been appointed assistant matron. She was trained at Birmingham Poor-law Infirmary, has been successively staff nurse at Christie Hospital, Manchester, charge nurse at her training-school, head nurse at Warwick Infirmary, and home sister, charge midwife, and night sister at the Portsmouth Workhouse Infirmary.

Maternity Hospital, Crewe.—Miss Florence Fox has been appointed matron. She was trained at the Essex County Hospital, where she was later assistant matron.

Naval Training Ship "Mercury," Hamble, Southampton.—Miss M. C. Harris, A.R.R.C., has been appointed sister-in-charge. She was trained at the West Kent General Hospital, Maidstone, has been staff nurse and sister at the Royal Infirmary, Leicester, sister at St. Anne's Convalescent Home, Bridlington Quay, and at the Desford Convalescent Home, Leicester, assistant matron of the Nurses' Institution, Stoke-on-Trent, sister T.F.N.S. at the North Evington Military Hospital, and assistant matron of the City of Westminster Infirmary. She has been housekeeping pupil at the Lord Mayor Treloar Hospital, Alton.

Royal Alexandra Hospital for Children, Brighton.—Miss Hilda Kidd has been appointed ward sister. She was trained at the Metropolitan Hospital and at the Children's Hospital, Carshalton, was sister at the Brighton, Hove, and Preston Dispensary, and out-patients' and night sister at the Royal Alexandra Hospital. Miss H. Wood has been appointed night sister. She was trained at Woolwich Infirmary, has been staff nurse at Victoria Hospital, Southend, and at the Children's Hospital, Carshalton, ward sister at Southwark Hospital, East Dulwich, and night sister at Gravesend Hospital.

Kilsyth Fever Hospital.—Miss A. R. Hay has been appointed matron. She was trained at the Royal Halifax Infirmary, has been sister at Midlothian County Fever Hospital, and matron of Montrose Fever Hospital.

Burnley Union Infirmary.—Miss E. A. Cook has been appointed night sister and Miss Turner and Miss G. Carter ward sisters. Miss Cook was trained at Greenwich Infirmary, and has been ward and night sister at Hastings Infirmary and maternity sister at Selly Oak Hospital. Miss Turner was trained at the Seamen's Hospital, Greenwich, and at the Royal Waterloo Hospital. Miss Carter was trained at Guy's Hospital, and has been ward sister at Camberwell Infirmary, casualty and theatre sister at the Queen's Jubilee Hospital, London, night sister and theatre sister at the Essex County Hospital, casualty and out-patient sister at Queen Mary's Hospital, night superintendent at Whipps Cross Hospital, and sister at the Royal National Hospital, Ventnor.

### The College of Nursing.

(Limited by Guarantee.)

### LONDON CENTRE.

(Secretary, Miss M. A. Bompas, 7 Henrietta Street, W. 1.)

### Unpaid Subscriptions.

The four hundred members of the London Centre who have still not paid their annual subscription, due April 1, are asked to forward it at once or to inform the Secretary that they do not wish to renew their membership.

### Centre Scholarship.

A monster Jumble Sale will be held on Saturday, October 15, at the Crossway Mission Hall, New Kent Road, S.E., to raise funds for the London Centre Scholarship. All College members are asked to begin to collect articles for it. Several members have kindly consented to receive contributions, among them being Miss Sheldon, 14 St. Thomas Street, S.E., and Miss Windly, Paddington Infirmary. The names of others can be obtained from Miss Bompas, London Centre Club, 7 Henrietta Street, W. 1, to whom also articles may be sent.

### YORKSHIRE CENTRE, LEEDS.

(Hon. Secretary, Miss Lindall, Hospital for Women and Children.)

About thirty members thoroughly enjoyed the Garden Party held at Seacroft Hospital, Leeds, on Saturday, July 30. Tennis and croquet were played after tea by some of the party, others were taken round the beautiful grounds of the hospital.

There will be no meeting in August, as many members are away on leave.

Members are reminded that the Centre subscription of 5s. was due on April 1, and those wishing to renew their membership should send their subscription at once. If they do not wish to remain members, will they let the Hon. Secretary know?

### Light Reading for the Holidays.

Messrs. Stanley Paul & Co. have just published half a dozen novels, by Cecil Adair, in the series known as the "Joy of Life" novels. Their titles are "Cantacute Towers," "The Sails of Life," "The Qualities of Mercy," "The Dean's Daughter," "Under the Incense Trees," and "Gabriel's Garden." The price of each is 2s. net; the print is clear, and the size of the book particularly handy for reading on the beach, by the roadside, or in the fields. Cecil Adair's readers, and they are numerous, should be happy in thus having these new novels within their reach, and those holiday-makers who have not read any of this writer's stories will, we have no doubt, hasten to avail themselves of the present opportunity.

August 13 1921] [Price Twopence

### The Hospital

**RATES OF SUBSCRIPTION** (post free, payable in advance).
Three months, 3/3; six months, 6/6; twelve months, 13/-. (Should the cost of printing and paper as well as increased postage, necessitate alteration of these rates, the period paid for will be reduced proportionately on unexpired subscriptions.) THE HOSPITAL "Index" to Volume LXIX., October 1920 to March 1921, is now ready, price 1/1 per copy, post free. Address The Manager, THE HOSPITAL, "The Hospital" Building, 28/29 Southampton Street, Strand, London, W.C. 2.

